# Supplementary material for: SARS-CoV-2 proteome microarray for global profiling of COVID-19 specific IgG and IgM responses
Source: Nat Commun. 2020 Jul 14;11:3581. doi: 10.1038/s41467-020-17488-8 (PMC7360742; doi:10.1038/s41467-020-17488-8)
Supplement: Supplementary file 2 — Supplementary Information [file 41467_2020_17488_MOESM2_ESM.pdf]

Supplementary Information

SARS-CoV-2 proteome microarray for global profiling of COVID-19 specific IgG and IgM responses

Jiang et al.

Table of content:

Supplementary Figure 1. The SARS-CoV-2 proteins included in this proteome microarray.
Supplementary Figure 2. The specificity of the microarray results.
Supplementary Figure 3. The overall SARS-CoV-2 specific IgG profiles of the 29 sera against the proteins with variant constructs and dilutions.
Supplementary Figure 4. The overall SARS-CoV-2 specific IgM profiles of the 29 sera against the proteins with variant constructs and dilutions.
Supplementary Figure 5. IgG response to S and N proteins.
Supplementary Figure 6. IgM Antibody response to S and N proteins.
Supplementary Figure 7. Significance analysis of microarray (SAM) plot for IgG responses against all the SARS-CoV-2 proteins on the microarray.

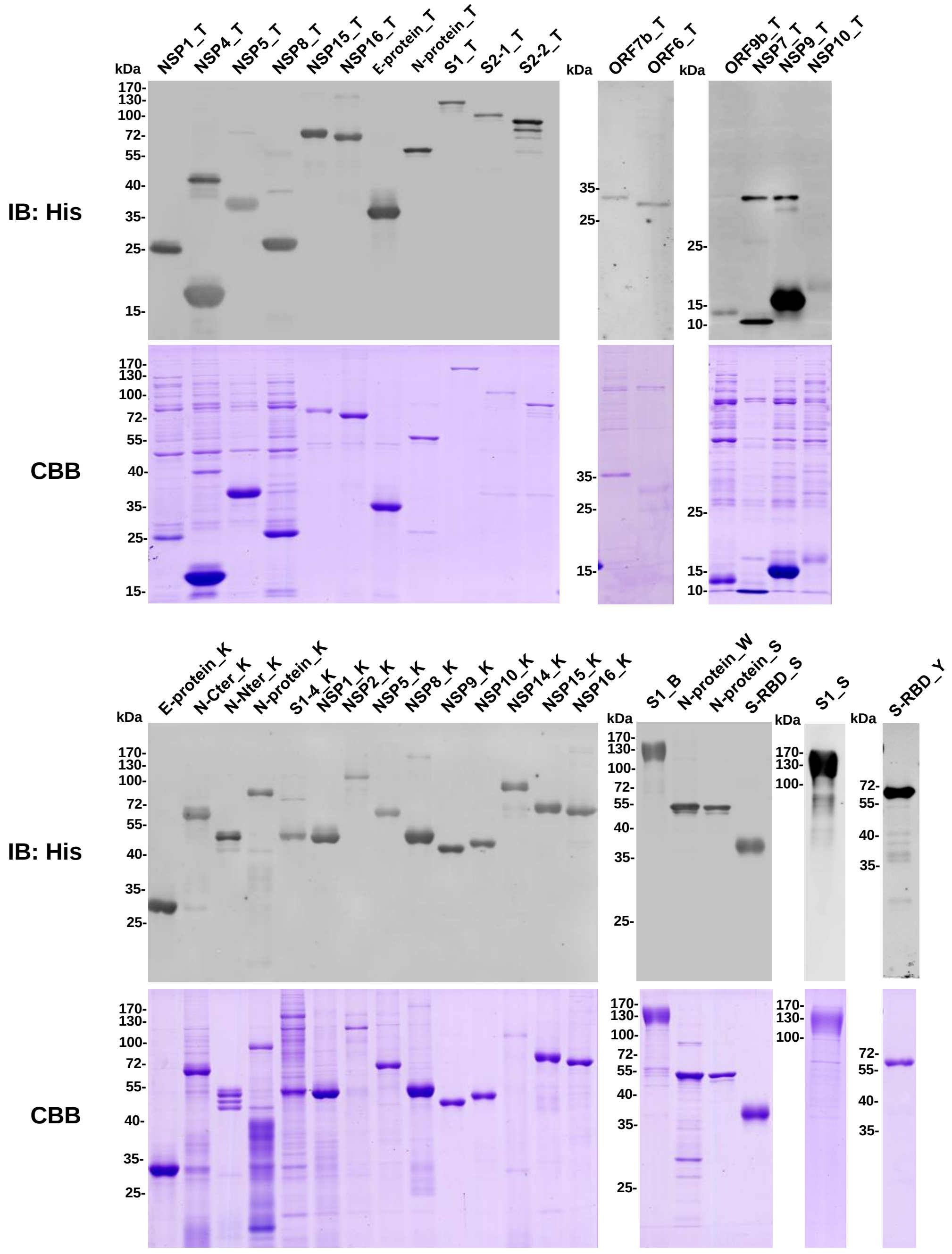

Supplementary Figure 1. The SARS-CoV-2 proteins included in this proteome microarray. The upper panel is western blotting with an anti-6xHis antibody. The lower panel is Coomassie staining. These proteins were prepared and collected from different sources. **NC**: negative conrol; **PC**: positive control; 0.1, 0.2, 0.25 and 0.5 indicate the concentration of these proteins for microarray printing. **T**: Tao Lab; **B**: Hangzhou Bioeast biotech. Co.,Ltd.; **K**: Healthcode Co., Ltd.; **S**: Sanyou biopharmaceuticals Co.,Ltd.; **W**: VACURE l Biotechnology Co.,Ltd. **Y**: Sino biological Co.,Ltd. **Expression system**: 1) **E.coli**: All proteins from Tao Lab (T), N Protein _S, N Protein_W; 2) **Cell-free**: All proteins from Healthcode Co., Ltd. (K), 3) **Mammalian**: S1_B, S1_S, S-RBD_S, S-RBD_Y. The SDS-PAGE was independently repeated for two times with similar results.

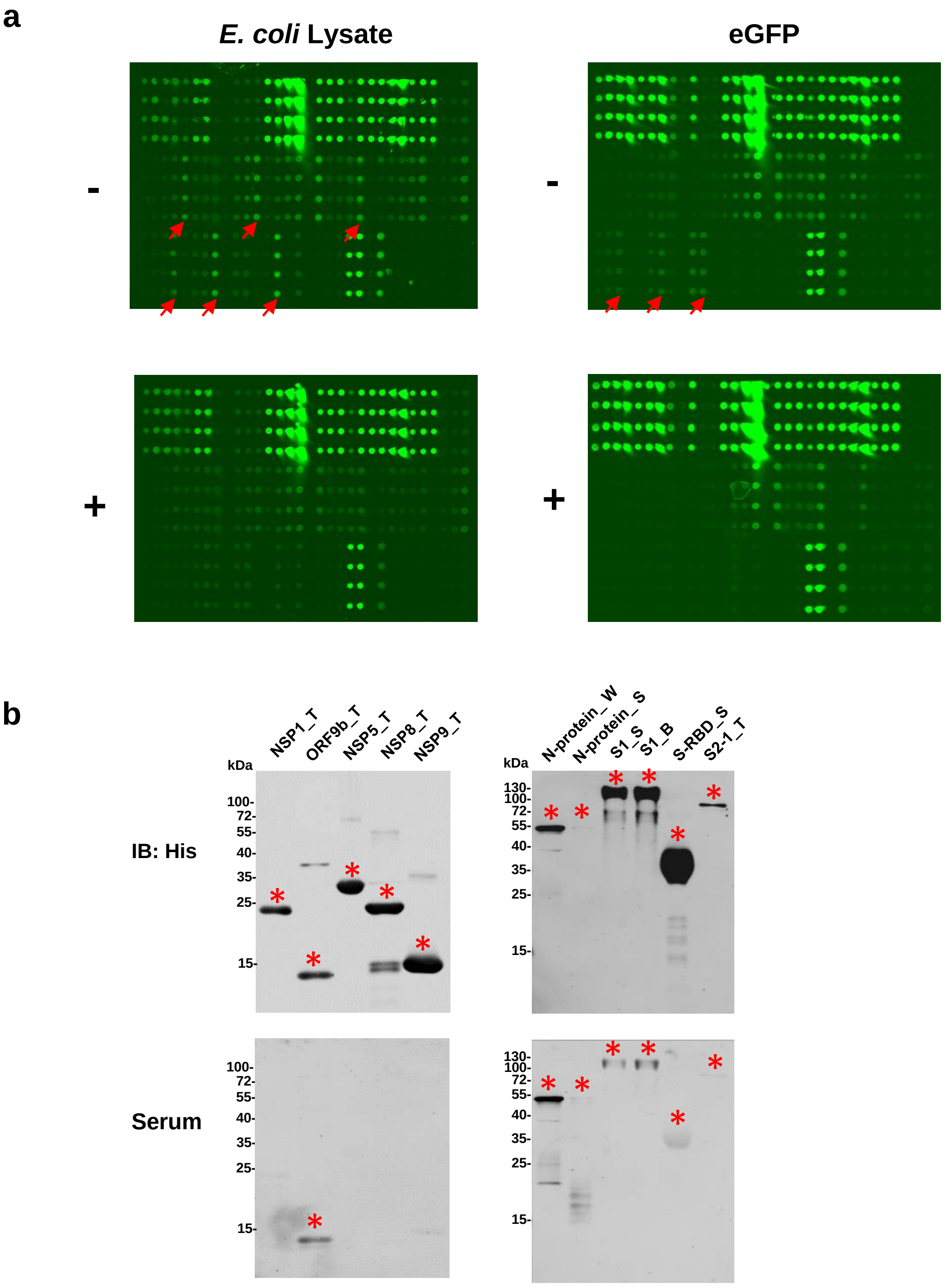

Supplementary Figure 2. The specificity of the microarray results. a Representative sub-arrays probed with serum of a COVID-19 convalescent. The non-specific bindings were reduced by the addition of *E. coli* lysate or eGFP in the incubation buffer. The red arrows indicate the proteins that could generate non-specific binding when the *E. coli* lysates or eGFP were not used. Red arrows indicate nonspecific binding. **b** The immunoblotting of selected proteins of SARS-CoV-2. The Up panel is western blotting with an anti-6xHis antibody. The low panel is western blotting with a serum sample of a COVID-19 convalescent, which showed positive binding to ORF9b on the microarray with besides to S protein and N protein. The red asterisks show the expected size of the protein. The immunoblotting was independently repeated by the same serum sample for two times with similar results. Red stars indicate the protein of expected size.

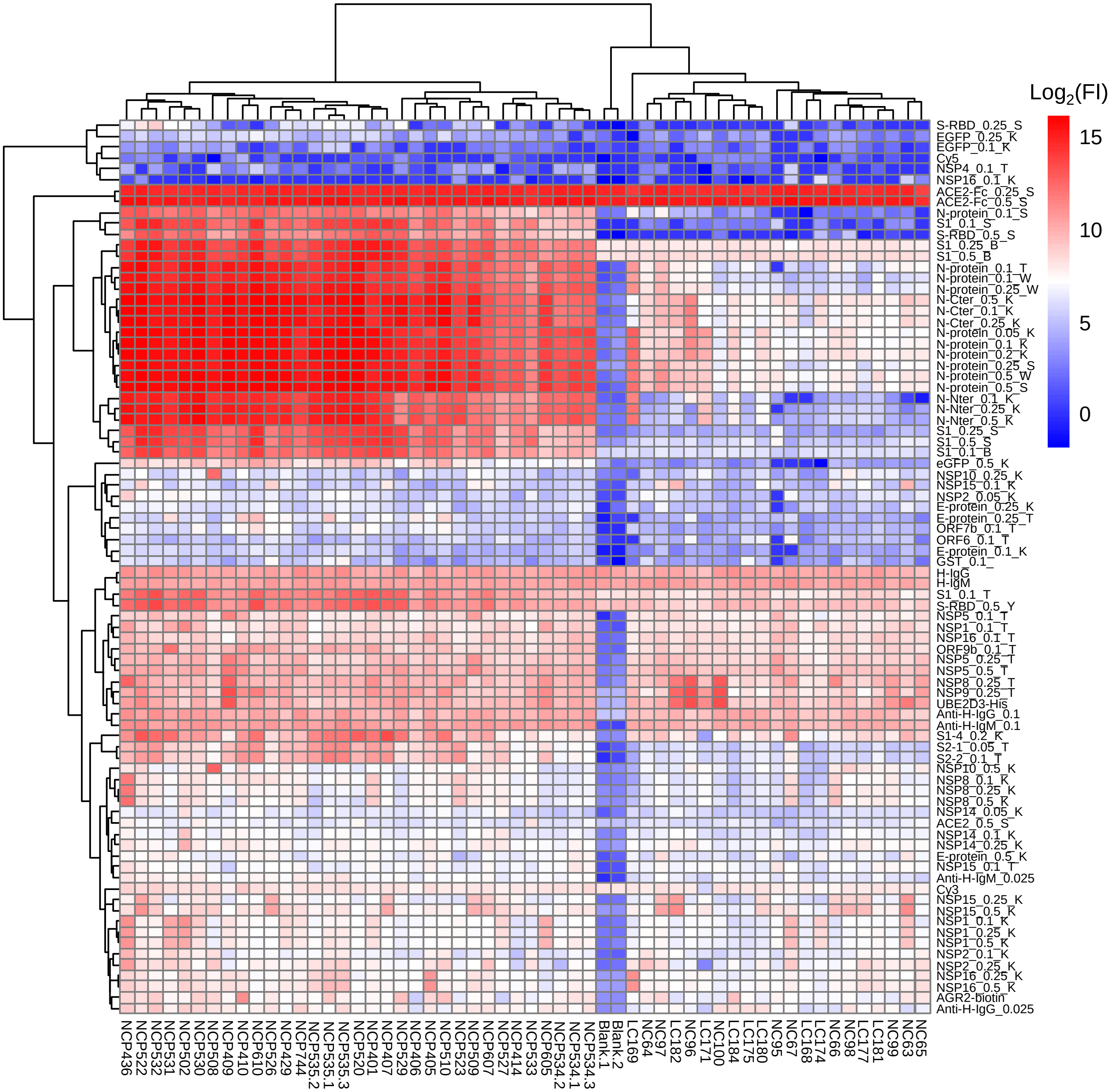

Supplementary Figure 3. The overall SARS-CoV-2 specific IgG profiles of the 29 sera against the proteins with variant constructs and dilutions. NC: negative control; PC: positive control; 0.1, 0.2, 0.25 and 0.5 indicate the concentration of these proteins for microarray printing.

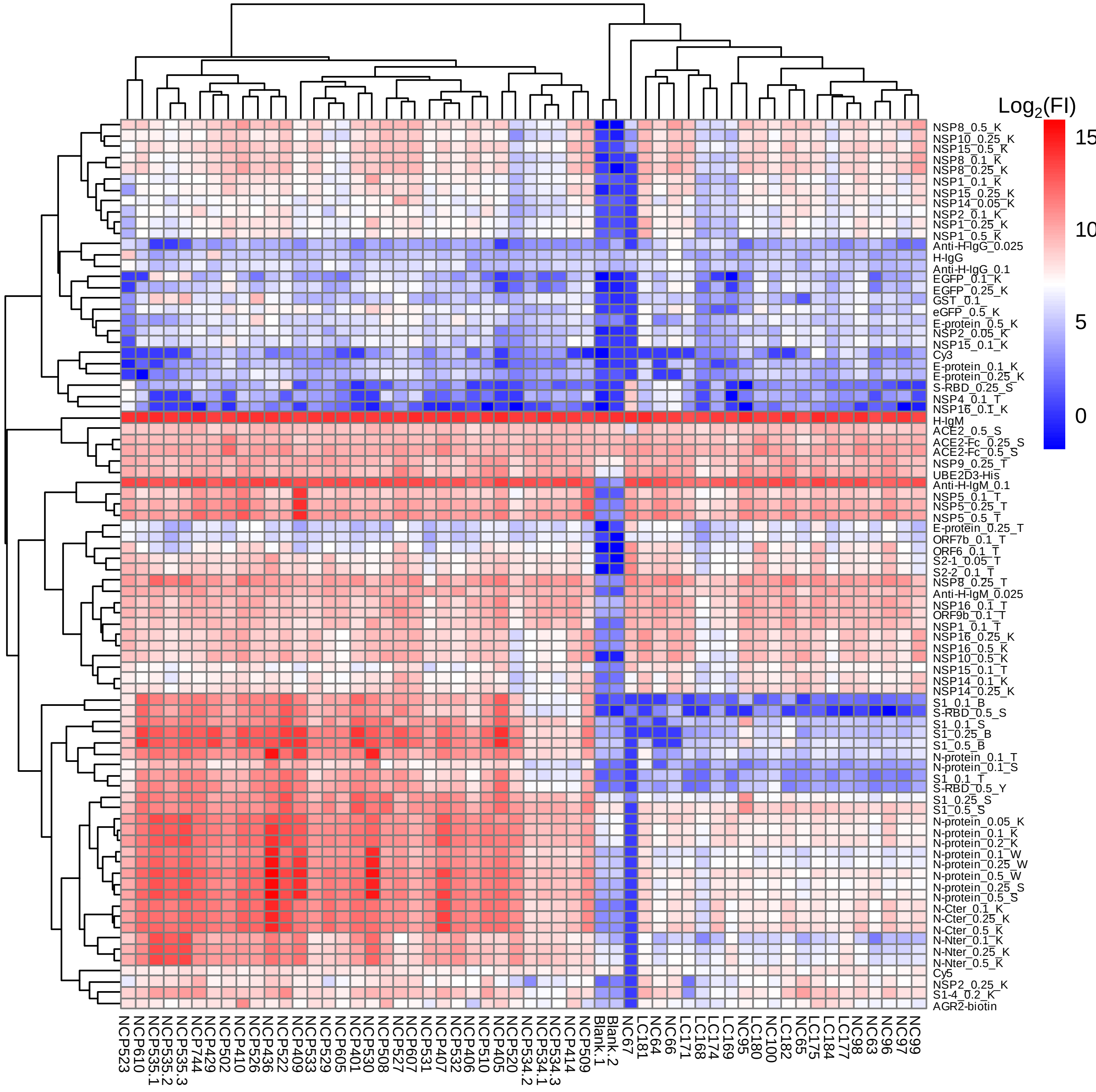

Supplementary Figure 4. The overall SARS-CoV-2 specific IgM profiles of the 29 sera against the proteins with variant constructs and dilutions. NC: negative control; PC: positive control; 0.1, 0.2, 0.25 and 0.5 indicate the concentration of these proteins for microarray printing.

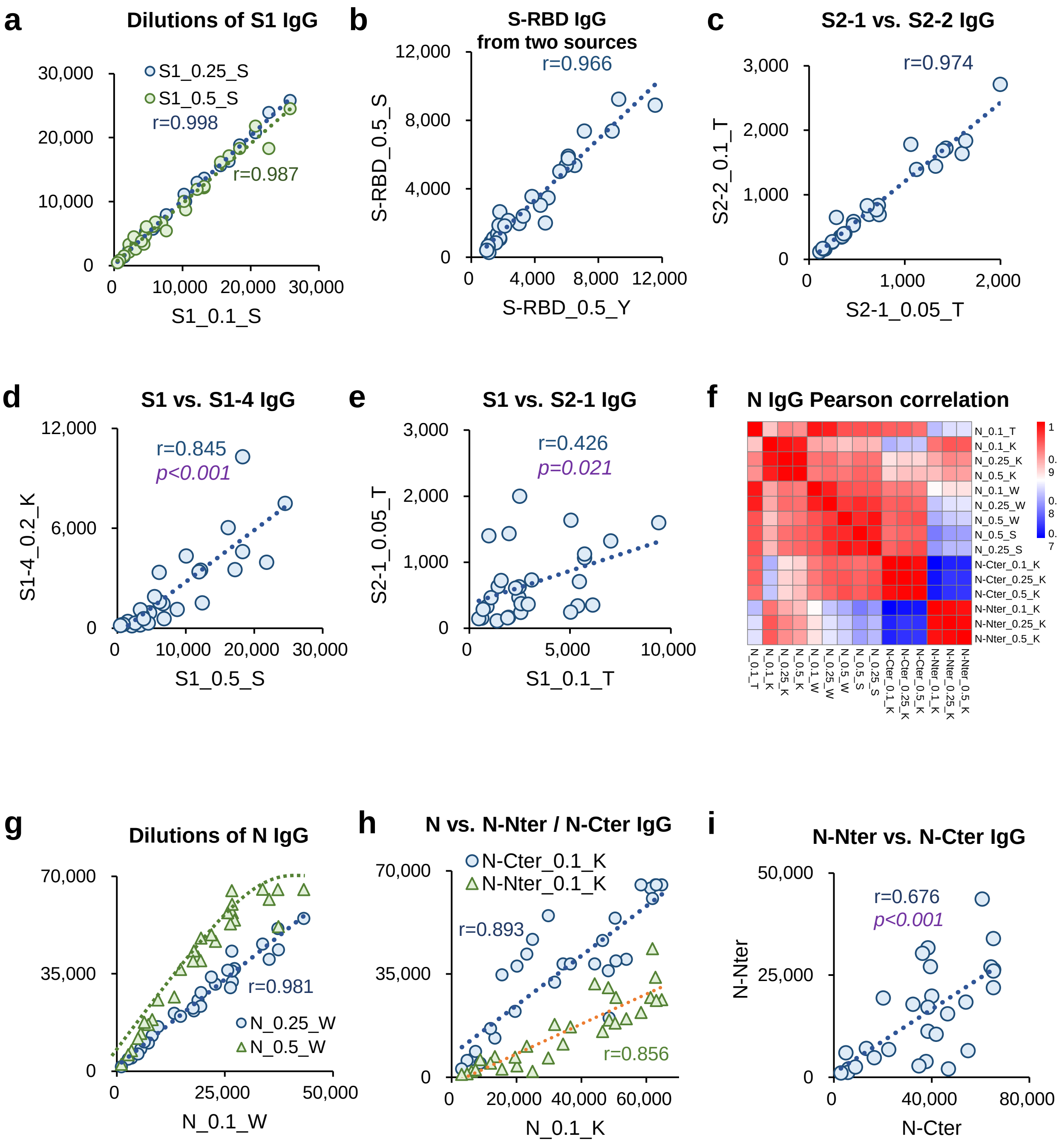

Supplementary Figure 5. IgG response to S and N proteins. a-e Correlations of the overall IgG responses among different dilutions of S1_S protein (**a**), two RBD proteins from different sources (**b**), S2-1 vs. S2-2 (**c**), S1 vs. S1-4 (**d**) and S1 vs. S2 (**e**). **f** Pearson correlation coefficient matrix of IgG responses among N proteins. **g-i** Correlations of the overall IgG responses among different dilutions of N protein_W (**g**), N-Nter/ N-Cter vs. N protein (**h**) and N-Cter vs. N-Nter (**i**). For **a-e**, **g-i**, each dot indicates one serum sample from the convalescent group (*n* =29). For **d**, **e** and **i**, *p* values were calculated by two-sided *t* test.

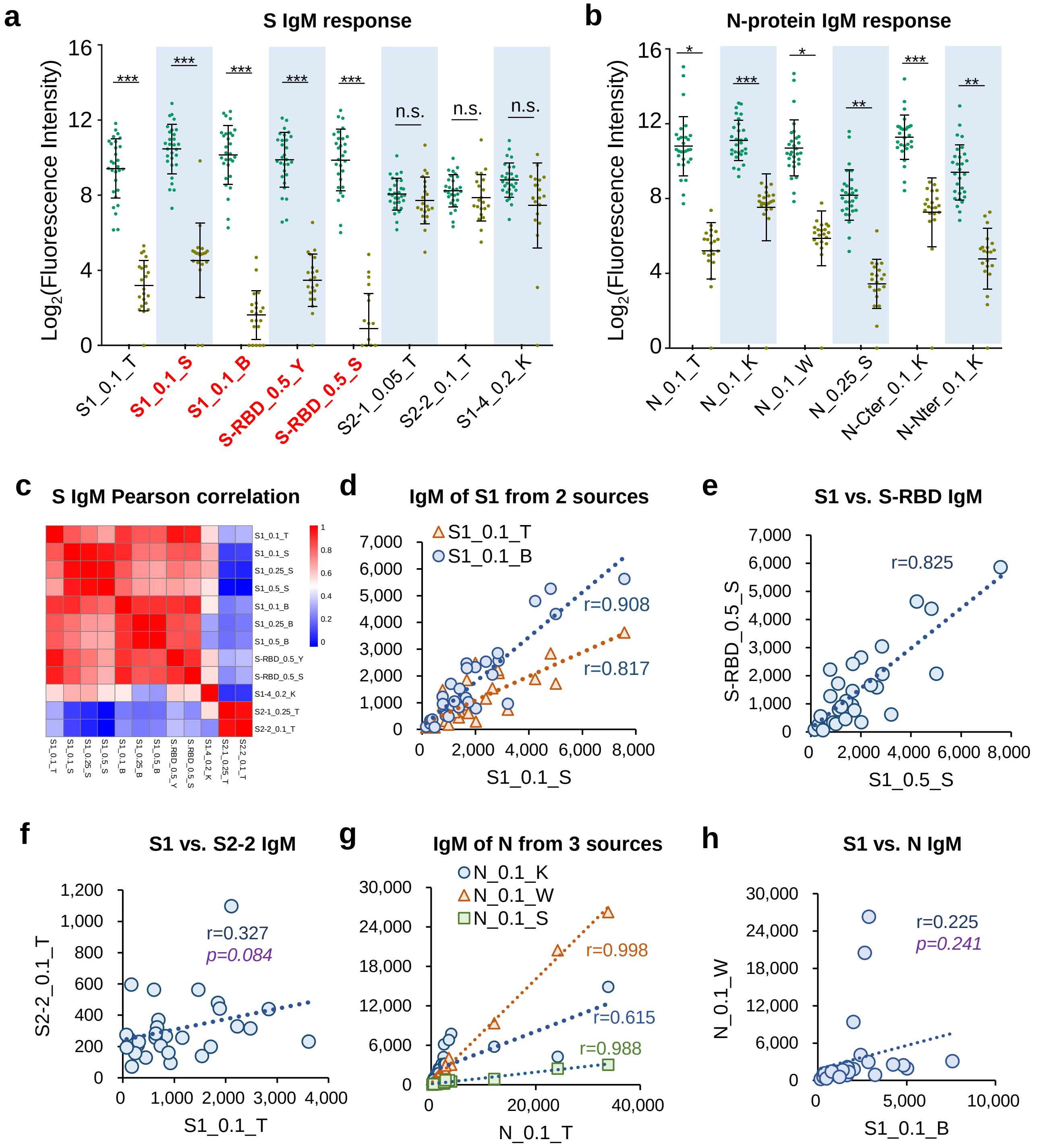

Supplementary Figure 6. IgM Antibody response to S and N proteins. a Box plots of IgM responses to S1 and S2 proteins. The proteins labeled with bold and red were overexpression in mammalian cell lines. **b** Box plots of IgM responses to N proteins. For **a-b**, each dot indicates one serum sample either from the patient group (green, *n* =29) or the control group (brown, *n* =21). Data are represented as boxplots where the middle line is the mean value. The upper and lower hinges are mean values +/- SD. *P* value was calculated by two-sided t test and *q* values were adjusted *p* values using BH method. ***, *q* <0.001; **, *q* <0.01; *, *q* <0.05; n.s., not significant. **c** Pearson correlation coefficient matrix of IgM responses among S1 and S2 proteins of different versions from different sources. **d-h** Correlations of the overall IgM responses among S1 proteins (**d**), S1 vs. RBD (**e**), S1 vs. S2 proteins (**f**), different N proteins (**g**) and N vs. S proteins (**h**). For **d-h**, each dot indicates one serum sample from the convalescent group (*n* =29). For **f** and **h**, *p* values were calculated by two-sided *t* test.

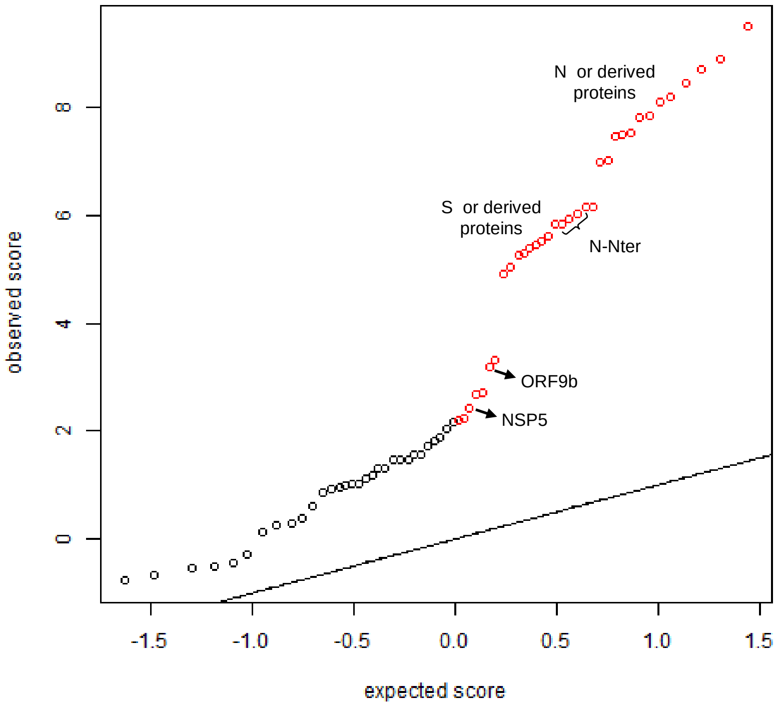

Supplementary Figure 7. Significance analysis of microarray (SAM) plot for IgG responses against all the SARS-CoV-2 proteins on the microarray. Red circles indicate the proteins with significant positive signals in patient group according to the SAM method. SARS-CoV-2 proteins (*n* = 65) on the microarray were examined.
