# Supplementary material for: SARS-CoV-2 proteome microarray for global profiling of COVID-19 specific IgG and IgM responses
Source: Nat Commun. 2020 Jul 14;11:3581. doi: 10.1038/s41467-020-17488-8 (PMC7360742; doi:10.1038/s41467-020-17488-8)
Supplement: Supplementary file 5 — Reporting Summary [file 41467_2020_17488_MOESM5_ESM.pdf]

nature research

Corresponding author(s): Sheng-ce Tao

Last updated by author(s): Jun 28, 2020

Reporting Summary

Nature Research wishes to improve the reproducibility of the work that we publish. This form provides structure for consistency and transparency in reporting. For further information on Nature Research policies, see Authors & Referees and the Editorial Policy Checklist .

Statistics

For all statistical analyses, confirm that the following items are present in the figure legend, table legend, main text, or Methods section.

n/a	Confirmed	
☐	☒	The exact sample size (*n*) for each experimental group/condition, given as a discrete number and unit of measurement
☐	☒	A statement on whether measurements were taken from distinct samples or whether the same sample was measured repeatedly
☐	☒	The statistical test(s) used AND whether they are one- or two-sided<br>*Only common tests should be described solely by name; describe more complex techniques in the Methods section.*
☐	☒	A description of all covariates tested
☐	☒	A description of any assumptions or corrections, such as tests of normality and adjustment for multiple comparisons
☐	☒	A full description of the statistical parameters including central tendency (e.g. means) or other basic estimates (e.g. regression coefficient) AND variation (e.g. standard deviation) or associated estimates of uncertainty (e.g. confidence intervals)
☐	☒	For null hypothesis testing, the test statistic (e.g. *F*, *t*, *r*) with confidence intervals, effect sizes, degrees of freedom and *P* value noted<br>*Give P values as exact values whenever suitable.*
☒	☐	For Bayesian analysis, information on the choice of priors and Markov chain Monte Carlo settings
☒	☐	For hierarchical and complex designs, identification of the appropriate level for tests and full reporting of outcomes
☐	☒	Estimates of effect sizes (e.g. Cohen's *d*, Pearson's *r*), indicating how they were calculated

Our web collection on statistics for biologists contains articles on many of the points above.

Software and code

Policy information about availability of computer code

Data collection	Data from microarray was collected by the software Genepix 6.1.
Data analysis	Pearson correlation coefficient between two proteins or indicators and the corresponding p-value was calculated by SPSS software version 22) under the default parameters. Cluster analysis was performed by pheatmap package in R 3.5.1(https://cran.r-project.org/web/packages/pheatmap/index.html). SAM was performed with "samr" package of R 3.5.1. The multiple linear regression was performed with the function “lm” from the “stats” package of the R 3.5.1. To make the coefficients in the regression model more comparable with each other, the values of all predictor vairables (x) have been normalized as follows: (x – min(x))/(max(x) – min(x)).

For manuscripts utilizing custom algorithms or software that are central to the research but not yet described in published literature, software must be made available to editors/reviewers. We strongly encourage code deposition in a community repository (e.g. GitHub). See the Nature Research guidelines for submitting code & software for further information.

Data

Policy information about availability of data

All manuscripts must include a data availability statement. This statement should provide the following information, where applicable:
- Accession codes, unique identifiers, or web links for publicly available datasets
- A list of figures that have associated raw data
- A description of any restrictions on data availability

The SARS-CoV-2 proteome microarray data are deposited on Protein Microarray Database (http://www.proteinmicroarray.cn) under the accession number PMDE241. Additional data related to this paper may be requested from the authors.

Field-specific reporting

Please select the one below that is the best fit for your research. If you are not sure, read the appropriate sections before making your selection.

☒ Life sciences ☐ Behavioural & social sciences ☐ Ecological, evolutionary & environmental sciences

For a reference copy of the document with all sections, see nature.com/documents/nr-reporting-summary-flat.pdf

Life sciences study design

All studies must disclose on these points even when the disclosure is negative.

Sample size	We screened sera from 29 convalescent patients, along with 21 controls. No sample size calculation was performed. Sample size was determined/limited by available number of samples. The maximum number of samples available was analyzed. Positive and negative control samples for assay development showed a clear difference in reactivity.
Data exclusions	No data were excluded.
Replication	All attempts at replication were successful. Specifically, high reproducibility of the protein microarray experiments was verified by triplicated test for two randomly selected samples. . Other samples were tested once.
Randomization	There was no separation of experimental groups in the study.
Blinding	Data collection and analysis were performed at the same time by the same batch of microarrays for all the samples.

Behavioural & social sciences study design

All studies must disclose on these points even when the disclosure is negative.

Study description	*Briefly describe the study type including whether data are quantitative, qualitative, or mixed-methods (e.g. qualitative cross-sectional, quantitative experimental, mixed-methods case study).*
Research sample	*State the research sample (e.g. Harvard university undergraduates, villagers in rural India) and provide relevant demographic information (e.g. age, sex) and indicate whether the sample is representative. Provide a rationale for the study sample chosen. For studies involving existing datasets, please describe the dataset and source.*
Sampling strategy	*Describe the sampling procedure (e.g. random, snowball, stratified, convenience). Describe the statistical methods that were used to predetermine sample size OR if no sample-size calculation was performed, describe how sample sizes were chosen and provide a rationale for why these sample sizes are sufficient. For qualitative data, please indicate whether data saturation was considered, and what criteria were used to decide that no further sampling was needed.*
Data collection	*Provide details about the data collection procedure, including the instruments or devices used to record the data (e.g. pen and paper, computer, eye tracker, video or audio equipment) whether anyone was present besides the participant(s) and the researcher, and whether the researcher was blind to experimental condition and/or the study hypothesis during data collection.*
Timing	*Indicate the start and stop dates of data collection. If there is a gap between collection periods, state the dates for each sample cohort.*
Data exclusions	*If no data were excluded from the analyses, state so OR if data were excluded, provide the exact number of exclusions and the rationale behind them, indicating whether exclusion criteria were pre-established.*
Non-participation	*State how many participants dropped out/declined participation and the reason(s) given OR provide response rate OR state that no participants dropped out/declined participation.*
Randomization	*If participants were not allocated into experimental groups, state so OR describe how participants were allocated to groups, and if allocation was not random, describe how covariates were controlled.*

Ecological, evolutionary & environmental sciences study design

All studies must disclose on these points even when the disclosure is negative.

Study description	*Briefly describe the study. For quantitative data include treatment factors and interactions, design structure (e.g. factorial, nested, hierarchical), nature and number of experimental units and replicates.*
Research sample	*Describe the research sample (e.g. a group of tagged Passer domesticus, all Stenocereus thurberi within Organ Pipe Cactus National Monument), and provide a rationale for the sample choice. When relevant, describe the organism taxa, source, sex, age range and any manipulations. State what population the sample is meant to represent when applicable. For studies involving existing datasets,*

	describe the data and its source.
Sampling strategy	*Note the sampling procedure. Describe the statistical methods that were used to predetermine sample size OR if no sample-size calculation was performed, describe how sample sizes were chosen and provide a rationale for why these sample sizes are sufficient.*
Data collection	*Describe the data collection procedure, including who recorded the data and how.*
Timing and spatial scale	*Indicate the start and stop dates of data collection, noting the frequency and periodicity of sampling and providing a rationale for these choices. If there is a gap between collection periods, state the dates for each sample cohort. Specify the spatial scale from which the data are taken*
Data exclusions	*If no data were excluded from the analyses, state so OR if data were excluded, describe the exclusions and the rationale behind them, indicating whether exclusion criteria were pre-established.*
Reproducibility	*Describe the measures taken to verify the reproducibility of experimental findings. For each experiment, note whether any attempts to repeat the experiment failed OR state that all attempts to repeat the experiment were successful.*
Randomization	*Describe how samples/organisms/participants were allocated into groups. If allocation was not random, describe how covariates were controlled. If this is not relevant to your study, explain why.*
Blinding	*Describe the extent of blinding used during data acquisition and analysis. If blinding was not possible, describe why OR explain why blinding was not relevant to your study.*

Did the study involve field work? ☐ Yes ☒ No

Reporting for specific materials, systems and methods

We require information from authors about some types of materials, experimental systems and methods used in many studies. Here, indicate whether each material, system or method listed is relevant to your study. If you are not sure if a list item applies to your research, read the appropriate section before selecting a response.

Materials & experimental systems

n/a	Involved in the study	
☐	☒	Antibodies
☐	☒	Eukaryotic cell lines
☒	☐	Palaeontology
☒	☐	Animals and other organisms
☐	☒	Human research participants
☒	☐	Clinical data

Methods

n/a	Involved in the study	
☒	☐	ChIP-seq
☒	☐	Flow cytometry
☒	☐	MRI-based neuroimaging

Antibodies

Antibodies used	Monoclonal Anti-His•Tag® produced in mouse, purified immunoglobulin, Merck Millipore, Cat.No.D48962, lot number 3256669, dilution 1:2000, clone name not applicable; Cy3-conjugated goat anti-human IgG and Alexa Fluor 647-conjugated donkey anti-human IgM were from Jackson ImmunoResearch, PA, USA (109-165-008 and 709-605-073, respectively)
Validation	From manufacturer's website:<br>The His•Tag® Monoclonal Antibody is a mouse monoclonal antibody (IgG1) directed against the His•Tag sequence encoded by many of the Novagen® expression vectors as well as many other commercially available vectors. The antibody recognizes five consecutive histidine residues regardless of the surrounding amino acid context. The His•Tag Monoclonal Antibody binds to N-terminal, C-terminal, and internal His•Tag sequences. This antibody also detects the recombinant marker bands in the Perfect Protein™ and Trail Mix™ Western Markers for convenient visualization of accurate internal standards on Western blots*. For lowest background in Western blotting applications, Alkali-soluble Casein is recommended as a blocking agent.<br>* Not recommended for HRP-based detection of Trail Mix Western Markers.<br>The specificity of the secondary antibodies have been fully validated previously in our published studies (Li, EbioMedicine, 2020, 102674)

Eukaryotic cell lines

Policy information about cell lines

Cell line source(s)	The study involved HEK 293T cells (from ATCC)
Authentication	Cell lines were not authenticated
Mycoplasma contamination	Cells were checked and didn't contain mycoplasma.

Commonly misidentified lines (See ICLAC register)	No commonly misidentfied cell lines were used.

Human research participants

Policy information about studies involving human research participants

Population characteristics	The cases involving COVID-19 convalescent patients which diagnosed and confirmed by nucleic acid test included 13 men and 16 women. Of the 29 cases, 3 were mild cases and 26 were moderate cases. The average time of days after onset is 22.3±5.4 days, the average hospital stay is 17.9±5.7 days and the average age is 42.3±13.8 years. The COVID-19 convalescent serum samples were collected during the period from 2020-1-25 to 2020-2-27. The cases involving control serum included 10 lung cancer patients and 11 health control. Lung cancer patients included 5 men and 5 women and the average age is 55.9±7.3 years. Health control included 6 men and 5 women and the average age is 45.1±12.9 years. The Lung cancer patient serum samples were collected in 2017 and health control serum samples were collected during the period from 2017 to 2018.
Recruitment	All patients were recruited with no bias in Foshan Fourth Hospital, Foshan, China. The sera of control group were per-exsiting.
Ethics oversight	The Institutional Ethics Review Committee of Foshan Fourth Hospital, Foshan, China approved this study (2020005) and the written informed consent was obtained from each patient. The control samples were from another project (Li, EBioMedicine, 2020, doi: 10.1016/j.ebiom.2020.102674) approved by the Institutional Ethics Review Committee of Ruijin Hospital, Shanghai JiaoTong University School of Medicine, Shanghai, China.

Note that full information on the approval of the study protocol must also be provided in the manuscript.
