# Supplementary material for: SARS-CoV-2 proteome microarray for global profiling of COVID-19 specific IgG and IgM responses
Source: Nat Commun. 2020 Jul 14;11:3581. doi: 10.1038/s41467-020-17488-8 (PMC7360742; doi:10.1038/s41467-020-17488-8)
Supplement: Supplementary file 6 — Description of Additional Supplementary Files [file 41467_2020_17488_MOESM6_ESM.pdf]

Title: Supplementary Data 1.
Description: Detailed information on the recombinant proteins prepared in this study.

Title: Supplementary Data 2.
Description: Statistical analysis of the IgG antibodies response against the SARS-CoV-2 proteins.
